# Using Self-Guided Treatment Software (*ePST*) to Teach Clinicians How to Deliver Problem-Solving Treatment for Depression

**DOI:** 10.1155/2012/309094

**Published:** 2012-11-14

**Authors:** James A. Cartreine, Trina E. Chang, Janette L. Seville, Luis Sandoval, John B. Moore, Shuai Xu, Mark T. Hegel

**Affiliations:** ^1^Department of Psychiatry, Brigham and Women's Hospital, Harvard Medical School, 1249 Boylston Avenue 3rd floor, Boston, MA 02215, USA; ^2^Harvard Medical School, Boston, MA 02215, USA; ^3^Department of Psychiatry, Massachusetts General Hospital, Harvard Medical School, Boston, MA 02114, USA; ^4^Department of Psychiatry, Dartmouth-Hitchcock Medical Center, Dartmouth Medical School, Lebanon, NH 03756, USA; ^5^Department of Educational Psychology, University of Texas at Austin, Austin, TX 72705, USA

## Abstract

Problem-solving treatment (PST) offers a promising approach to the depression care; however, few PST training opportunities exist. A computer-guided, interactive media program has been developed to deliver PST electronically (*ePST*), directly to patients. The program is a six-session, weekly intervention modeled on an evidence-based PST protocol. Users are guided through each session by a clinician who is presented via hundreds of branching audio and video clips. Because expert clinician behaviors are modeled in the program, not only does the *ePST* program have the potential to deliver PST to patients but it may also serve as a training tool to teach clinicians how to deliver PST. Thirteen social workers and trainees used *ePST* self-instructionally and subsequently attended a day-long workshop on PST. Participants' PST knowledge level increased significantly from baseline to post-*ePST* (*P* = .001) and did not increase significantly further after attending the subsequent workshop. Additionally, attending the workshop did not significantly increase the participants' skill at performing PST beyond the use of the *ePST* program. Using the *ePST* program appears to train novices to a sufficient level of competence to begin practicing PST under supervision. This self-instructional training method could enable PST for depression to be widely disseminated, although follow-up supervision is still required.

## 1. Introduction

Effective treatments have been developed for depression, including antidepressant medications and psychotherapies. Many patients prefer counseling or psychotherapy to taking medications, if it is available [1], and matching treatment (medication or therapy) to patient preference has been demonstrated to improve outcomes, independent from depression severity [2, 3]. Unfortunately, many mental healthcare providers lack training in evidence-based treatments [4] or fail to implement them properly [5], making evidence-based psychotherapy unavailable to many persons with depression in the United States. One way to increase access to evidence-based psychotherapies is to develop more cost-efficient and accessible training methods.

Problem-solving treatment (PST; also known as problem-solving therapy) has emerged as an effective treatment for depression [6–8]. The basis of PST is that enhancing problem-solving skills and attitudes and working to solve concrete problems in one's life can reduce depression. PST breaks the problem-solving process into steps and teaches participants to go through these steps systematically and effectively, targeting parts of the process that are particularly challenging for depressed patients. PST has been found to produce outcomes comparable to antidepressant medications [9, 10] and better than treatment as usual [11, 12]. 

PST has great potential for dissemination because it can be delivered by mental health as well as nonmental health specialists in a wide range of healthcare settings, including primary care [13]. Healthcare professionals, such as nurses and social workers are trained in a one- to two-day seminar that includes didactics, demonstrations, and roleplay with feedback [14]. They are then supervised on PST by a recognized expert over the course of three to five cases. For each case, the supervisor listens to and critiques a recording of three PST sessions, sometimes providing this supervision via the telephone.

 Automating PST training could improve the ability to further disseminate PST and potentially result in cost savings. Although the supervision portion of the training requires the involvement of a live clinician, it is conceivable that some or all of the workshop could be automated. Self-instructional training is not a new concept; self-instructional books have existed for centuries and self-instructional videos for decades. These could be good starting points to learn PST. However, it has been demonstrated that the more interactive a training experience is, the more effective it is [15–18]. Since the skill at hand, PST, is a highly interactive process, developing a highly interactive self-guided training approach should enhance PST skill building.

### 1.1. *ePST* Software

An interactive media-based computer program has been developed to provide PST electronically (*ePST*) in an entirely automated manner [19]. In this self-help program, users are guided through six sessions of PST by a master clinician (M. T. Hegel) who is an expert in the intervention and conveys the “nonspecific” characteristics of effective therapists, such as warmth, genuineness, compassion, and the ability to provide support when the patient experiences setbacks [20]. Users interact with the program by entering text via the keyboard or by clicking on answers to questions, and hundreds of branching audio and video clips are used to tailor the program to users' inputs. The program tracks the user's depression via the Patient Health Questionnaire-9 (PHQ-9; [21]) and the on-camera host provides feedback and recommendations. The program reviews the user's work across sessions and across problems to provide guidance on how to improve his or her problem-solving success, through the use of failure analyses [22–24] algorithms. Although the program was originally developed for astronauts to treat their own depression on long-duration space missions, it was designed for use by the public. 

Because the program models best practices for providing PST, it may be useful not only as an intervention but also as a teaching tool, to demonstrate how to deliver PST. The purpose of this study was to obtain pilot data on the feasibility and efficacy of using this program to teach PST to therapists unfamiliar with the intervention. Primary research questions addressed were as follows.

1. Is use of *ePST *associated with change in knowledge of the steps and process of PST?
2. Is live training associated with change in skill at performing PST, for persons who have already been trained via *ePST*?

Secondary research questions were as follows.

1. Is use of *ePST* associated with change in self-efficacy for performing PST-specific skills, and for doing counseling in general?
2. How acceptable and easy to use is *ePST* for training?

## 2. Methods

### 2.1. Participants

The subject population consisted of clinical staff and trainees from a human services organization in Framingham, MA, with no prior training in PST. All participants volunteered for the study and received no compensation for participation. Training was offered to all clinicians and trainees working at the site.

### 2.2. Procedure

The study was approved by the Massachusetts General Hospital's Institutional Review Board, and consent to participate in the study was implied by voluntary completion of the study questionnaires. Receiving training was not contingent on participation in the evaluation component, and participants were not required to participate as part of their employment.

Training consisted of participants using the *ePST* program for four sessions *as if they were patients*. Participants were encouraged to work on real problems in their own lives via *ePST* as a means of learning how PST works. They were also asked to view the on-camera therapist as a model for delivering PST. Although a full course of treatment via *ePST* involves six sessions, the investigators judged *a priori* that using the program for only four sessions would provide sufficient exposure to the model for trainees to learn PST. At no point in this study did the participants interact with actual patients using the PST approach.

Assessments were conducted at three time points and trainings were conducted at two points. At baseline (before beginning training), participants completed a test of their knowledge of PST and a measure of self-efficacy to do counseling (regarding both general and PST-specific skills). Following completion of their training using *ePST,* participants again completed questionnaires on their knowledge of PST and self-efficacy as well as the acceptability and usability of the computer program. They also participated in audiotaped roleplay sessions as a PST therapist treating a standardized patient (a research assistant trained to portray a depression patient). After the workshop, participants completed the knowledge and self-efficacy questionnaires for the third time and then conducted another taped roleplay session with a different standardized patient. Three standardized patient cases of similar difficulty were written for the roleplay. Subjects were randomly assigned to different cases for their roleplay sessions. 

### 2.3. Measures

**Knowledge of PST**

This was a primary measure, which consisted of one open-ended essay-format question: “Please describe the process of problem-solving therapy in detail, including all steps and the criteria for successfully completing each one.” Essays were scored using criteria developed for the study (see Appendix A). The possible range of scores was 0 to 120, with higher scores indicating greater knowledge. The essays were rated by M. T. Hegel and J. L. Seville, blind to the time point of each essay.

**Skill Implementing PST**

The standardized patient roleplay was a primary measure. The audiotaped roleplay sessions were scored using the Problem-Solving Treatment in Primary Care Adherence and Competence Scale (PST-PAC) [25, 26] which evaluates trainees' performance on nine dimensions of PST, each on a six-point scale (0–5; worst to best performance) (see Appendix B). Because each standardized patient was being seen for an initial PST session, the first rating dimension, “Defining the outcome” (of the previous session's action plan) was not included. Therefore, participants were rated on dimensions two through eight of the PST-PAC. The possible range of scores was 0 to 40, with higher scores indicating better performance.

Scoring was performed by J. A. Cartreine and J. L. Seville, who were blind to the time point of each roleplay. One of the raters (J. L. Seville) had been trained in the use of the PST-PAC previously; the other (J. A. Cartreine) was trained to rate roleplays by rating audio recordings of sample cases.

**Self-Efficacy for Implementing PST and Doing Counseling, in General**

The *PST and Counseling Self-Estimate Inventory *(PCSEI) was created for this study, it consists of 30 items that measure different aspects of a therapist's perception of his or her ability to perform specific PST skills and general counseling skills (see Appendix C). It was adapted from the Counseling Self-Estimate Inventory (CSEI) [27]. To reduce the length of the measure, some items from the CSEI less relevant to the study were dropped, while several new items pertinent to performing PST-specific skills were added, using similar language. Items were scored on a six-point scale (1 = “strongly disagree”; 6 = “strongly agree”), with a possible range of 30 to 180 for the total measure, a range of 16 to 96 for the “PST-specific” subscale and 14 to 84 for the “General” subscale. Some items are reverse scored to avoid acquiescence bias; higher scores reflect higher self-efficacy.

**Acceptability of Using *ePST* to Learn PST**

The Program Acceptability Questionnaire (PAQ) was written for this study and consisted of six questions on various aspects of the acceptability of the PST computer program, such as how much the program helped the trainee learn PST and whether the subject would recommend its use to other prospective trainees (see Appendix D). Items were rated on a six-point scale as above (1 = “strongly disagree”; 6 = “strongly agree”).

**Usability of the *ePST* Program**

The System Usability Scale (SUS) [28, 29] was used to assess ease of using the software. The SUS is a ten-item questionnaire that is widely used for assessing software usability, such as whether a system is excessively complex or cumbersome to use, or whether the trainee needs technical support to use it. Statements about the software are rated on a six-point scale (0 = “strongly disagree”; 6 = “strongly agree”).

### 2.4. Analytic Methods

**Score Calculations**

Scores for the PST-PAC were obtained using the following procedures: for each standardized patient interview, all eight scores given by each rater were averaged, yielding two scores (one for each rater). For items where the raters' scores differed by more than one point, the raters discussed the item and arrived at a consensus score for that item. Then, the average was taken of the two raters' scores, yielding one final score per standardized patient interview.

PST knowledge essays were scored on 27 items that map onto seven subscales, which correspond to the steps of PST. Because the seven subscales contain different numbers of items, an average of the scores for each subscale, for each rater, was calculated. The seven scores were then summed, yielding one score per essay for each rater. Finally, an average was calculated between scores awarded by each rater, to obtain a full-scale score for each participant on each essay written.

Self-efficacy scores were calculated by summing the responses to individual items (with some questions reverse scored). Additionally, subscales were calculated by summing the items measuring general self-efficacy and those measuring PST-specific self-efficacy.

**Analyses**

Due to the small sample size, nonparametric analyses were used to answer the research questions. The Wilcoxon signed ranks test was used to compare skill levels between Assessment 2 and 3 (no skill test was administered in Assessment 1). The same test was also used to gauge change in knowledge and self-efficacy at Assessment 1 compared to 2, and Assessment 2 compared to 3. Because multiple comparisons were made, a Bonferroni correction was used, which set the *P* cutoff for significance at *P* = .0125 for the primary outcomes of skill and knowledge. The Bonferroni correction was not used for the secondary outcome of self-efficacy.

Descriptive statistics were used to characterize the sample and results of the SUS and PAQ. Because the PAQ was created for this study, an insufficient quantity of data has been collected to support a factor analysis and the reporting of a composite score. Therefore, results on this questionnaire are reported by item.

## 3. Results

Thirteen participants (11 female and 2 male) enrolled, with a mean age of 37.5 (±12.9); 11 self-identified as Caucasian and the other 2 as racial minorities. Six were licensed clinical social workers, with an average of 2 years in practice (±1.4, range 2 to 6 years); the other 7 were social work graduate students. No participants who inquired about the study subsequently refused to participate or dropped out. 

Interrater reliability for both the essay and roleplay scoring was calculated using percent agreement between raters. For the skill measure, interrater agreement per item (defined as agreement on the rating plus or minus 1 point) between the two raters averaged 79.6%. To increase concordance between raters, consensus scores (as described in the methods section) were obtained for items in which raters differed by >1 point. For the knowledge (essay) measure, the percent agreement (within one point) between the raters across items was 87.5%. Because agreement between raters on the knowledge measure was high from the outset, a consensus process was not needed to improve concordance. 

Summary results of the knowledge, skill, self-efficacy, and usability measures are presented in Table 1.

### 3.1. Primary Outcomes

Knowledge scores significantly increased from pretraining to post-using *ePST *(*Z* = 3.18, *P* = .001) but did not increase significantly more after attending the live workshop (*Z* = 1.363, *P* = .173). No significant difference was found in skill level after using *ePST* versus after the live workshop, using either nonparametric (Wilcoxon signed ranks test *Z* = −2.040; *P* = .041) or parametric (*t* = −2.346; *df* = 12; *P* = .037) statistics. This suggests that the workshop did not substantially increase the knowledge or skill of the participants beyond what they learned using the *ePST* program. On an item-level analysis of the roleplay ratings, only skill at helping patients identify pros and cons of solutions changed before to versus after attending the live workshop. On this item, before attending the workshop (but after completing *ePST*) the median score was 2.5 on a scale of 0 to 5 (range .5 to 4.5; mean of 2.5 ± 1); and after the workshop, the median increased to 3.5 (range .5 to 5.0; mean 3.81 ± .9; Wilcoxon signed ranks test *Z* = −2.599; *P* = .009). Also, across outcome measures and time points, there were no significant correlations between participants' knowledge, skill, or self-efficacy levels.

**Figure 1:**
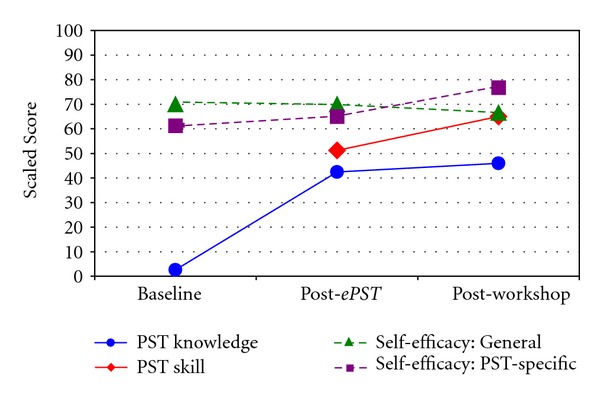
Scaled scores for skill, knowledge, and self-efficacy. Scaled Score = Median converted to percent of maximum possible score.

To facilitate comparisons between knowledge and skill levels, scaled scores of 0 to 100 were calculated by converting the median to the percentage of the maximum score possible on each scale (see Figure 1). Both knowledge and skill were near the midpoint after using *ePST* (42.46 and 51.25, resp.) and remained near that point after completing the workshop (45.97 and 65).

### 3.2. Secondary Outcomes

**Self-Efficacy for PST and Counseling, Overall**

Five items were unanswered in the PCSEI data set. Values for these missing data points were imputed by calculating the mean of the participant's responses to the other items from the same subscale on that administration of the PCSEI. Composite self-efficacy scores, which included all items on the PCSEI, remained stable from pretraining to post-*ePST* training to post-live workshop, although there was a significant increase in composite self-efficacy from pre-training to post-workshop (*P* = .028). The Wilcoxon signed ranks test *Z*-score for self-efficacy at pretest compared to post-*ePST* was −1.014; *P* = .311 (*t* = −1.365; *df* = 12; *P* = .199) and the *Z*-score for post-*ePST *compared to post-live training was −1.751; *P* = .080 (*t* = −1.977; *df* = 12; *P* = .071). On the PST-specific subscale, there was a significant improvement from post-*ePST* and post-live workshop training (*Z*-score = −2.033; *P* = .042). No significant difference was found for the subscale of general counseling self-efficacy between pretest and post-*ePST *training, or between post-*ePST *and post-live workshop training.

### 3.3. Usability and Acceptability

 The median SUS score was 67.5 (range 60 to 87.5; mean = 69.23 ± 7.8) on a scale of 0 to 100. On the PAQ, the medians for all but one item were 5 (means ranged from 4.54 to 5.23) on a scale of 0 to 6 (see Table 2).

## 4. Discussion

PST is an evidence-based intervention for depression; however, few providers have training in it. A novel approach to teaching PST is the use of a computer-automated treatment, *ePST*. Users are guided through it via branching videos to simulate the experience of being in treatment with a master clinician (M. T. Hegel). As such, best practices for delivering PST are modeled and the program may serve as a training tool for practitioners, in addition to a treatment for patients. 

Thirteen persons with no prior experience delivering PST (6 licensed clinical social workers and 7 social work graduate students) used the *ePST *interactive media program for four sessions over the course of two weeks. They were instructed to work on a real problem in their own lives, to make the training more meaningful. After the self-guided instruction, all 13 attended a standard PST training workshop presented by a veteran PST trainer (also M. T. Hegel), which is the standard method to train new practitioners. 

It was found that live training does not add knowledge or skill beyond what *ePST *provides. Trainees were assessed on knowledge at three points: once at baseline, again after using *ePST *for four sessions, and again after completing the live day-long workshop. They were also assessed on PST skill via roleplays with standardized patients after completing *ePST *and again after completing the workshop. Knowledge about how to conduct PST went from a near-zero level (1.5 on a scale of 0 to 35) at baseline to a significantly higher level after using *ePST *(mean score 14.1) and did not significantly improve following the workshop (mean score 16.7). 

PST skill was not assessed at baseline due to logistical constraints; however, it is likely that it would also have been very low before training, since none of the subjects had received prior training. After completing 4 sessions of *ePST*, mean skill was 19.7 (on a scale of 0 to 40) and was 25.2 following the live workshop. This change was not statistically significant. Composite self-efficacy for performing PST skills and for doing counseling in general gradually increased from baseline to post-live training. This appears to be due to a significant improvement in self-efficacy for performing PST skills after the workshop and one standardized patient interview. Therefore, confidence in one's ability to conduct PST increased significantly as a result of the live workshop and/or standardized patient interview, even though actual skill and knowledge did not.

The only specific areas of improvement found after completing the workshop were knowledge and implementation of decision-making guidelines (i.e., evaluation of pros and cons in problem solving). Both skill and knowledge regarding decision-making guidelines improved following the workshop, suggesting that *ePST *could be strengthened in this regard. No significant change in either direction was noted for other content areas following the live workshop.

A key ingredient in clinical training is supervision while treating actual patients, and it is unrealistic to expect that use of a self-instructional training program or a one-day workshop would produce fully competent PST clinicians. However, after using *ePST, *social workers and social work trainees possessed a sufficient level of skill to begin providing PST under supervision. As such, *ePST* may be a convenient and low-cost alternative to the in-person PST workshops that are the standard of practice. As the number of PST practitioners increases, the number of persons with sufficient experience to serve as supervisors will also increase. Moreover research has already supported PST supervision via telephone [12], meaning the supervisor need not be in the same room—or the same time zone—as the trainee. Together, *ePST *plus telephone-based supervision could support the diffusion of PST throughout the medical and mental health communities. 

Regarding the usability of *ePST*, an analysis of all published studies that used the SUS to evaluate software of all types found that the mean score was 70.14 [28]. The mean SUS score for *ePST* was 69.23; however, this may underestimate the program usability. Because *ePST* is designed for weekly sessions, to run the program more than once per week (as was done in this study), participants had to manually advance the system date on their computers, which can be challenging. A training version of *ePST* could readily overcome this limitation. 

Acceptability of *ePST* appeared high. That said results of the Program Acceptability Questionnaire, written for this study, may have been subject to response set, as the means and standard deviations were very similar between items. For example, on a scale of 1 to 6, the mean of “I would recommend the program to a colleague” was 4.54 (±1.13); similarly, “I would rather do training in a live workshop than with the computer” was also 4.54 (±1.05). 

*ePST* appears to be only the second computer program to evaluated the potential of a computer-automated treatment to train clinicians, and this appears to be the first study to use a behavioral outcome measure (i.e., standardized patient roleplays). The researchers identified one other computer-automated treatment program that has been used to teach clinicians how to perform a skill. *FearFighter*, a UK program for these treatment of panic and phobias [30], was used to teach the process of exposure therapy in two studies [31, 32]. Both studies used written measures to assess instructional gains, and both concluded that educational gains were equivalent to classroom training. 

This study advances the literature on self-instructional training of therapists, and of clinicians in general, by using an e-therapy program as a training tool to teach clinicians an evidence-based treatment. Research on self-directed/computer-automated/online training has reported mixed training outcomes, likely because the pedagogies of the tools vary widely, from passively viewed didactics to written manuals to interactive programs [33]. Highly interactive training experiences, such as using *ePST*, have been demonstrated to produce superior outcomes regarding therapists' skill and actual behavior in the implementation of evidence-based treatments [33].

### 4.1. Limitations and Future Directions

This study has several limitations, including the mixed educational background of the sample and the lack of a comparison group design, which limit generalizability. Without a comparison group, it is impossible to say for certain what trainees learned from *ePST* versus from the live workshop. This study also had a relatively small sample size; however, we have noted that training studies often enroll fewer participants than treatment studies. Additionally, the use of standardized patients is a well-accepted measure of clinician skill [34, 35]; however, participants' performance might have been different with actual patients. Confidence in these results would be strengthened if interrater reliability of participant skill (in the roleplays) were higher. Finally, trainees may have learned from participating in the roleplay (the first of which happened after the post-*ePST* assessment and before the workshop); this effect might have artificially inflated the estimates of learning from the workshop, thus leading to an underestimate of the full effect of *ePST* training.

Regarding modifications to the *ePST *software, many trainees suggested the inclusion of a modeling video demonstrating the use of PST with a patient. Such a video has already been recorded and can be incorporated into a training version of *ePST*. Also, there exists a wealth of print training material for PST that could augment the computer-based training. Finally, supervision of new PST therapists is a necessary component of PST training [25, 26] and is unlikely to be automated.

Several national and statewide initiatives are implementing evidence-based treatments for depression and could benefit from cost-effective training, such as those by the National Network of Depression Centers [36], The University of Washington's Improving Mood: Promoting Access to Collaborative Treatment (IMPACT) [37] and its Program to Encourage Active, Rewarding Lives for Seniors (PEARLS) [38] programs, and Depression Improvement Across Minnesota, Offering a New Direction (DIAMOND) [39]. *ePST *opens the door to a new training method that could promote the mass dissemination of PST among healthcare providers in order to improve patient access to care and ultimately reduce the impact of depression in the population.

## Figures and Tables

**Table 1:** Assessments of knowledge, skill, self-efficacy, and *ePST* program usability.

	Pre-training			Post-*ePST* (computerized) Training			Post-live training		
	Median (range)	Mean ± SD	Scaled score (0 to 100)^a^	Median (range)	Mean ± SD	Scaled score (0 to 100)^a^	Median (range)	Mean ± SD	Scaled score (0 to 100)^a^
Knowledge of PST	0.9 (0 to 6.45)	1.53 ± 2.04	2.57	14.86^b^ (3.33 to 24.49)	14.06 ± 6.74	42.46	16.09 (8.74 to 24.63)	16.74 ± 5.17	45.97
Skill implementing PST	—	—	—	20.5 (7 to 28)	19.65 ± 6.70	51.25	26 (11 to 34)	25.23 ± 7.16	65.00
Self-efficacy (Composite)	126 (106 to 162)	129.54 ± 14.64	64.00	133 (112 to 156)	134.31 ± 13.81	67.33	145^c^ (106 to 168)	143.31 ± 17.36	76.67
Self-efficacy (General)	63 (54 to 76)	63.31 ± 6.29	70.00	65 (52 to 73)	64.23 ± 6.10	70.00	66 (54 to 78)	66.54 ± 8.23	74.29
Self-efficacy (PST-specific)	65 (50 to 86)	66.23 ± 9.59	61.25	68 (52 to 86)	70.08 ± 9.21	65.00	77^d,e^ (52 to 90)	76.77 ± 10.52	76.25
Usability of *ePST *	—	—	—	67.5 (60 to 87.5)	69.23 ± 7.8	—	—	—	—

^a^Median converted to percent of maximum possible score.

^b^Compared to pre-training (*P* = 0.001).

^c^Compared to pre-training (*P* = 0.028).

^d^Compared to pretraining (*P* = .023).

^e^Compared to post-*ePST* (*P* = .042).

**Table 2:** Program Acceptability Questionnaire (scale range 0 to 6).

Item	Mean ± SD	Median (range)
I felt comfortable using the PST program for training	5.23 ± 0.73	5 (4 to 6)
Doing training using this program was acceptable to me	4.92 ± 0.76	5 (4 to 6)
Using the program helped me understand how to do PST	4.69 ± 0.75	5 (4 to 6)
I enjoyed using the program to learn PST	4.62 ± 1.12	4 (3 to 6)
I would rather do training in a live workshop than with the computer	4.54 ± 1.05	5 (3 to 6)
I would recommend this program to a colleague who is interested in learning how to do PST	4.54 ± 1.13	5 (2 to 6)

## Appendices

### A. PST Knowledge

#### A.1. Problem-Solving Treatment Knowledge Assessment

Authors James A. Cartreine, Brigham and Women's Hospital/Harvard Medical School, Boston, MA. Janette L. Seville, Geisel School of Medicine at Dartmouth, Hanover, NH. Trina E. Chang, Massachusetts General Hospital/Harvard Medical School, Boston, MA. Mark T. Hegel, Geisel School of Medicine at Dartmouth, Hanover, NH.

Instructions In the space below, please answer the following essay question. You have 10 minutes.
*Please describe the process of problem-solving therapy in detail, including all steps and the criteria for successfully completing each one.*

Scoring Criteria Assess whether the written response addresses each element below, on a scale of 0–5.****

**Table:**

0	1	2	3	4	5
Very Poor	Poor	Borderline	Satisfactory	Good	Very Good

**Table:**

Number	Element	Subscale
(1)	Problem should be observable	Problem
(2)	Problem should be under client's control	Problem
(3)	Goal should be behavioral (something client does)	Goal
(4)	Goal should be general (more than one way to reach it)	Goal
(5)	Goal should be observable (or countable)	Goal
(6)	Goal should be achievable (or short term, i.e., 2 weeks)	Goal
(7)	Goal should not just be the converse of the problem	Goal
(8)	Brainstorming should include multiple ideas/solutions	Brainstorming
(9)	Do not prejudge solutions	Brainstorming
(10)	Pros are identified for each idea/solution (or what makes each unique)	Solutions
(11)	Multiple cons are assessed for each idea/solution	Solutions
(12)	Effort to implement solution is assessed	Solutions
(13)	Time to implement solution is assessed	Solutions
(14)	Cost of solution is assessed	Solutions
(15)	Need for other people to implement solution is assessed	Solutions
(16)	Negative impact on others is assessed	Solutions
(17)	One or more solutions must be chosen	Solutions
(18)	Action plan can be implemented soon	Action plan
(19)	Action plan is step by step, detailed	Action plan
(20)	Action plan includes Who, What, Where, and When are the steps to be taken	Action plan
(21)	Anticipate obstacles and plan around them	Action plan
(22)	Plan B's (backup plans) are created	Action plan
(23)	Enjoyable activities should be scheduled for each week	Pleasant events
(24)	Clinician checks whether action plan was implemented and goal was reached	Progress check
(25)	Clinician checks client's satisfaction with his or her effort	Progress check
(26)	Clinician helps clients troubleshoot how to improve their problem solving	Progress check

### B. PST Skill

#### B.1. Problem-Solving Treatment in Primary Care Therapist Adherence and Competence Scale

Version 8-13-09

Authors Mark T. Hegel, Ph.D., Geisel School of Medicine at Dartmouth, Hanover, NH. Laurence Mynors-Wallis, M.D., University of Southampton, UK.

Rater Instructions For each item, assess the therapist on a scale of 0–5 and record the rating on the line next to the item number.****

**Table:**

0	1	2	3	4	5
Very Poor	Poor	Borderline	Satisfactory	Good	Very Good

****

1. Evaluating the outcome
  Review of all current tasks
  Praise success
  Exploration of failure
  Rate Satisfaction and Mood
  Reinforce PST-PC Model
  Review previous problem areas
2. Defining the problem
  Specific, feasible problem chosen
  Described in objective terms
  Problem explored, clarified
  Identify barriers
3. Establishing a realistic goal
  Goal is objective
  Described in behavioral terms
  Goal is achievable
  Goal is general
  Follows directly from problem statement
  Addresses barriers identified in problem
4. Generating solutions
  Prime for brainstorming
  Brainstorming facilitated
  Solutions from patient
  Withhold judgment
5. Implementing Decision-Making Guidelines and Choosing the Solution(s)
  Consider “pros” and “cons” for one's self/others
  Rate each theme
  Compare solutions
  Solution(s) satisfies the goals
  Negative impact is limited
6. Implementing the preferred solution(s)
  Specific tasks identified
  Realistic behavior requirements
  Plan B
  Plan pleasant activities for the week
7. Process tasks
  Clear demarcation of PST-PC stages
  Kept session near a 30-minute timeframe
  Cue and review for stages
  Summarize process at end of session
  Facilitate a positive problem orientation
  Facilitate independence in guiding PST process
8. Communication and interpersonal effectiveness
  Facilitates communication (supportive vocalizations/nonverbals)
  Use of patient's own language and phrases
  Warm/confident/professional
  Tactful limiting of peripheral and unproductive discussion
9. Global rating
  How would you rate the problem-solving therapist overall in this session? (does not need to approach a mathematical average of previous eight items)

### C. Self-Efficacy

#### C.1. PST and Counseling Self-Estimate Inventory

Authors Based on Larson et al. [27]. (PST-specific questions added by the authors.)

Instructions This is not a test. There are no right or wrong answers. Rather, this is an inventory that attempts to measure how you think you behave doing problem-solving therapy. Please respond to the items as honestly as you can to most accurately portray how you think you behave as a PST therapist. Do not respond with how you wish you could perform each item, or think you might in the future. Rather, answer in a way that reflects your actual estimate of how you perform as a counselor at the present time.****

**Table:**

1	2	3	4	5	6
Strongly Disagree					Strongly Agree

**Table:**

	Item	Subscale
(1)	I can effectively redirect clients who choose to work on problems over which they have limited control	PST
(2)	I am likely to impose my values on the client during the interview	General
(3)	When I initiate the end of a session, I am positive it is in a manner that is neither abrupt nor brusque, and I end sessions on time	General
(4)	I can help clients construct action plans that meet the criteria of problem-solving therapy	PST
(5)	I feel that I will not be able to respond to the client in a nonjudgmental way with respect to the client's values, beliefs, and so forth	General
(6)	I feel that I can respond to the client in an appropriate amount of time (neither interrupting the patient nor waiting too long to respond)	General
(7)	I anticipate that the type of response I use at a particular time may not fit with the problem-solving therapy approach	PST
(8)	I can help clients with the brainstorming step of problem-solving therapy	PST
(9)	I feel confident that I have resolved conflicts in my personal life so that they will not interfere with my counseling abilities	General
(10)	I feel that I have enough fundamental knowledge to do effective problem-solving therapy	PST
(11)	I am able to respond in a helpful way when clients report that they have not worked on a problem since last session	PST
(12)	I may not be able to maintain the intensity and energy level needed to produce client confidence and active participation	General
(13)	I am not sure that when doing problem-solving therapy I will express myself in a way that is natural without deliberating over every response or action	PST
(14)	I am confident that I can conduct problem-solving therapy, adhering to the guidelines set out in the PST therapy manual	PST
(15)	My assessments of client problems may not be as accurate as I would like them to be	PST
(16)	I am confident that I can help patients define their problems in a manner suitable for problem solving	PST
(17)	I do not feel I possess a large enough repertoire of techniques to deal with the different problems my client may present	General
(18)	I am uncomfortable about dealing with clients who appear unmotivated to work toward their goals	PST
(19)	I have difficulty dealing with clients who do not verbalize their thoughts during the counseling session	General
(20)	I am unsure how to deal with clients who appear noncommittal and indecisive	General
(21)	I am an effective counselor with clients of a different socioeconomic status	General
(22)	I am unsure how to lead my client to work toward concrete goals	PST
(23)	I am confident that I can assess my client's readiness and commitment to work on a given problem	PST
(24)	When working with ethnic minority clients I am confident that I will be able to bridge cultural differences in the counseling process	General
(25)	I am confident that I can help my patients evaluate the pros and cons of their solutions and choose a feasible solution in an efficient and helpful manner	PST
(26)	I am confident that I can support the client in choosing his or her own problems on which to work	PST
(27)	I feel I may give advice	General
(28)	In working with culturally different clients, I have a difficult time viewing situations from their perspective	General
(29)	I anticipate having difficulty helping clients write goal statements that meet all of the problem-solving therapy criteria	PST
(30)	I am afraid that I may not be able to effectively relate to someone of lower socioeconomic status than me	General

### D. Acceptability

#### D.1. Program Acceptability Questionnaire

Author James A. Cartreine, Brigham and Women's Hospital/Harvard Medical School, Boston, MA.

Instructions Please read each item carefully and circle a number to show how much you agree or disagree with the statement.****
**Table:**

1	2	3	4	5	6
Strongly Disagree					Strongly Agree

****

1. I felt comfortable using the PST program for training
2. Doing training using this program was acceptable to me
3. Using the program helped me understand how to do PST
4. I enjoyed using the program to learn PST
5. I would rather do training in a live workshop than with the computer
6. I would recommend this program to a colleague who is interested in learning how to do PST
